# Effects of multigenerational imidacloprid and thiamethoxam stress on metabolism and physiology of *Aphis glycines* Matsumura (Hemiptera: Aphididae)

**DOI:** 10.1371/journal.pone.0271069

**Published:** 2022-07-08

**Authors:** Aonan Zhang, Wenjing Zhou, Dongxue Wu, Lanlan Han, Kuijun Zhao

**Affiliations:** College of Agriculture, Northeast Agricultural University, Harbin, Heilongjiang, PR China; University of Carthage, TUNISIA

## Abstract

The soybean aphid, *Aphis glycines* Matsumura (Hemiptera: Aphididae), a primary pest of soybean, poses a severe threat to soybean production. In this study, the 4^th^ instar nymphs were exposed to the LC_50_ and LC_30_ of imidacloprid and thiamethoxam from F0 to F4 generations to evaluate the activities of peroxidase, pyruvate kinase, and trehalase using microassay. We found that peroxidase and pyruvate kinase activities in soybean aphids increased rapidly, first to peak and then decreased slowly generation by generation under imidacloprid and thiamethoxam stress. In contrast, the trehalase activity was significantly decreased in F1 to F5 generations when treated with the LC_50_ and LC_30_ and imidacloprid and thiamethoxam compared to control. In addition, the Enzyme-Linked Immunosorbent Assay (ELISA) was used to monitor the changes in molting and juvenile hormone expressions of the soybean aphids in each generation (F1-F5). The expression of juvenile hormone in soybean aphids was increased significantly in each generation under continuous stress of imidacloprid and thiamethoxam LC_50_ imidacloprid and LC_50_ thiamethoxam inhibited the expression of molting hormones in soybean aphids of each generation. LC_30_ imidacloprid or LC_30_ thiamethoxam significantly stimulated the expression of molting hormone in the 1^st^ and 2^nd^ instar nymphs in each generation. In this paper, the differences in antioxidant regulation, energy metabolism intensity, and hormone expression of multi-generation soybean aphids were monitored under continuous stress of imidacloprid and thiamethoxam. Our results revealed the effects of continuous insecticide stress on the main endogenous substances. Further, they clarified the regulation rules of resistance in soybean aphids, providing a reference for efficient control with imidacloprid and thiamethoxam.

## Introduction

The soybean aphid, *Aphis glycines* Matsumura (Hemiptera: Aphididae), is native to Asia and has been mainly distributed in soybean-growing areas in the Far East [1]. *Aphis glycines* was first detected in Wisconsin (USA) in 2000. Since then, this insect has spread to the North Central and Midwestern United States. Although the damage from *A*. *glycines* to soybeans was rarely devastating in Asia, it was considered a primary pest in North America [2–4]. At present, the application of insecticides remains an effective management approach for preventing soybean aphid outbreaks [5]. Neonicotinoids were the most effective in controlling soybean aphids owing to their unique action mode [6]. Neonicotinoids could selectively act on the central nervous system of insects as agonists of nicotinic acetylcholine receptors (nAChRs), blocking normal nerve signal transduction and causing abnormal stimulation spasm, paralysis, and death of pests [7]. In 2010, the global market share of neonicotinoids had exceeded 25% [8]. Among them, imidacloprid and thiamethoxam had a good control effect on soybean aphids, and were widely used [6, 8–10]. Imidacloprid and thiamethoxam were representative products of the first and second generations of neonicotinoids, respectively. Imidacloprid is a chloronicotinoid compound with heterocyclic groups with 6- chloropyridine -3- methyl. Thiamethoxam belongs to the sub-class of thianicotinyl, which has a novel 1, 3, 5-oxadiazine ring [11]. Jiang et al. [12] reported that thiamethoxam was more toxic than imidacloprid to the eye gnat *Liohippelates collusor* Townsend (Diptera: Chloropidae). Ding et al [13] showed that thiamethoxam had a better control impact on corn thrips *Frankliniella williamsi* Hood (Thysanoptera: Thripidae) than imidacloprid under the same dosing conditions [12, 13]. Our previous study also confirmed that imidacloprid had a stronger impact on the growth and reproduction of the soybean aphid population than thiamethoxam at the same concentration [14]. Although they are both neonicotinoids, their heterocyclic structures might lead them to have different pest control effects [11].

The effect of different insecticides on soybean aphids was directly reflected in the change of control effect, survival rate, development time, and fecundity parameters. It was also indirectly expressed in the regulation of physiological metabolism by soybean aphids. Metabolic regulation of soybean aphids was not as easy to observe as the former. Researchers often monitored the changes in the expression of key physiologically active chemicals to determine the changes in the metabolic environment of pests [15, 16]. At present, some researchers have done relevant research, as Wang et al. [15] who evaluated the metabolic detoxification capacity and resistance accumulation degree of pests by monitoring the changes in peroxidase activity. Birnbaum et al. [16] assessed pest resistance to insecticide injury by monitoring changes in superoxide dismutase, catalase, and peroxidase activity [17]. Similarly, Sheng et al. [18] found that the LC_30_ of phoxim and cypermethrin could stimulate the activities of peroxidase and glutathione S-transferase. This study also suggested that the changes in enzyme activity might be related to the potential detoxification mechanism of pests [18]. Studies have focused on related enzymes involved in the glycolysis pathway and changes in the juvenile hormone and molting hormone titer. Ge et al. [19] confirmed the prominent role of glycolysis in the resistance of insects to insecticide stress. Pyruvate kinase, a rate-limiting enzyme in the glycolysis pathway, catalyzed the formation of the second ATP, which was crucial for reproductive growth and stress resistance [19]. Trehalase was also involved in glycolysis, hydrolyzing trehalose into glucose [20]. Wegener et al. [21] found that insect survival decreased with the decrease of trehalase activity. In addition, The embryo development of insects was affected by pyruvate kinase and regulated by juvenile and molting hormones [22–24]. In insects, juvenile and molting hormones were present at minute levels and were continuously synthesized and degraded. Both were crucial resources for determining the reproductive potential of populations and regulating resistance [25]. In this study, we comprehensively monitored the activities of peroxidase, pyruvate kinase, and trehalase and the expressions of juvenile hormone and molting hormone in the 4^th^ instar nymphs from F1 to F5 under continuous stress of imidacloprid and thiamethoxam. To evaluate the differences in antioxidant regulation, energy metabolism intensity, and hormone expression in the multi-generation soybean aphids under insecticide stress. These results demonstrated the effects of continuous insecticide stress on the main endogenous substances of multi-generation soybean aphids. Further, they clarified the regulation rules of resistance in different generations of soybean aphids. It was of great significance to monitor resistance changes in the field population, delay the accumulation of resistance in soybean aphids, prolong insecticide’s service life, and effectively control soybean aphids with imidacloprid and thiamethoxam.

## Materials and methods

### Laboratory soybean aphid population

The laboratory strain of *A*. *glycines* used in this study was originally collected from a soybean field in Harbin, Heilongjiang Province, China, in 2016. This strain had been cultured in the laboratory without exposure to any insecticide. The soybean plants (Dongnong 252) were grown in pots (15 cm in diameter × 17 cm in depth), with six plants per pot to maintain *A*. *glycines* strain at 25 ± 1°C with 65%–70% relative humidity and 14-:10-h (L:D) photoperiod in Northeast Agricultural University, China. One-third of old aphid-infested soybean plants were removed and replaced with fresh plants twice a week. This prevented the accumulation of excessive honeydew and sooty mould, and ensured that the aphids had a uniform soybean plant for feeding [26].

### Chemical agents

Water dispersible granules of insecticides (70% imidacloprid (trade name Yashijing) and 50% thiamethoxam (trade name Aketai)) were purchased from North China Pharmaceutical Group Corporation, Hebei, China and Shaanxi Thompson Biotechnology Co., Ltd., Shaanxi, China, respectively. Phosphate Buffer Saline (PBS) was purchased from Beijing Boaotuo Technology Co., LTD., Beijing, China. Peroxidase assay kit, pyruvate kinase assay kit, trehalase assay kit, and Coomassie bright blue protein content assay kit was purchased from Suzhou Keming Biotechnology Co., LTD., Jiangsu, China. The juvenile hormone of the insect ELISA kit and the molting hormone of insect ELISA kit was purchased from Jiangsu Enzyme Labeled Biotechnology Co., LTD., Jiangsu, China.

### Concentration-response bioassay of imidacloprid and thiamethoxam

The insecticidal stock solution was prepared with 1% acetone and diluted to different concentrations with 0.05% (v/v) Triton X-100 distilled water. The concentration-response bioassays were conducted with the 4^th^ instar nymphs using the leaf dip method recommended by the Insecticide Resistance Action Committee (IRAC; http://www.irac-online.org/resources/methods.asp). Seven concentrations of imidacloprid (30.05, 19.95, 10.05, 5.64, 3.47, 2.59, and 1.72 mg a.i./L) and thiamethoxam (56.45, 30.62, 18.35, 10.80, 6.38, 4.64, and 2.98 mg a.i./L) were prepared according to the preliminary experiment. Fresh soybean leaf discs were dipped in the insecticide concentrations for 10 seconds and then placed on paper towels at room temperature for air drying. The discs were dipped in distilled water containing 0.05% (v/v) Triton X-100 and 1% acetone for control treatments. The treated leaf discs were placed in a Petri dish (6 cm in diameter) with an agar medium. The 4^th^ instar nymphs were introduced to all treated leaf discs. Handling details (insecticide, concentration, and date) were recorded for each Petri dish. Each concentration was replicated three times. Twenty 4^th^ instar nymphs were used for each replicate. Mortality was determined after 24 h of exposure. Aphids that were upside down or not moving when pushed gently with a soft brush were considered dead.

### Determination of the activities of peroxidase, pyruvate kinase and trehalase of 4^th^ instar nymphs in each generation (F1-F5)

The LC_50_ and LC_30_ concentrations of imidacloprid and thiamethoxam were prepared in distilled water containing 0.05% Triton X-100. Insecticide exposure was carried out, as discussed above. After 24 hours, the surviving 4^th^ instar nymphs of F0 generation were transferred to insecticide-free leaf discs. The leaf discs were changed every 24 hours. Thirty soybean aphids that grew to the 4^th^ instar nymphs of the F1 generation were selected and placed in 1.5 mL Eppendorf (EP) tubes. Each treatment was repeated three times. All tubes were cooled by liquid nitrogen and stored at -80°C. The chemical treatment of 4^th^ instar nymphs of F1 to F4 generations and collection of samples were the same as discussed above. The 4^th^ instar nymphs of F5 generation developed from the surviving soybean aphids of F4 generation were collected without any insecticide exposure. All tubes stored at -80°C were then placed in a plastic box (10 cm × 15 cm × 10 cm) containing ice cubes. Add 300 μL PBS to each EP tube and thoroughly grind the soybean aphid using a plastic rod. The EP tube was placed in the plastic box for 2 minutes and centrifuged at 10000 r·min^-1^ for 10 minutes. The supernatant was transferred to a new EP tube. The peroxidase, pyruvate kinase, and trehalase of the 4^th^ instar nymphs in each generation were monitored by microassay using Suzhou Coming biological enzyme activity assay kit.

### Determination of the titers of molting hormone and juvenile hormone of the soybean aphids in each generation (F1-F5)

The LC_50_ and LC_30_ concentrations of imidacloprid and thiamethoxam were prepared in distilled water containing 0.05% Triton X-100. Insecticide exposure was carried out, as discussed above. After 24 hours, the surviving 4^th^ instar nymphs of F0 generation were transferred to insecticide-free leaf discs. The leaf discs were changed every 24 hours. Thirty 4^th^ instar nymphs that grow to the F1 generation were selected and placed in 1.5 mL Eppendorf (EP) tubes. Each treatment was repeated three times. All tubes were cooled by liquid nitrogen and stored at -80°C. The chemical treatment of the 4^th^ instar nymphs of F1 to F4 generations and collection of samples were the same as discussed above. The 4^th^ instar nymphs of F5 generation developed from the surviving individuals of F4 generation were collected without any insecticide exposure. All tubes stored at -80°C were then placed in a plastic box (10 cm × 15 cm × 10 cm) containing ice cubes. Add 300 μL PBS to each EP tube and thoroughly grind the soybean aphid using a plastic rod. The EP tube was placed in the plastic box for 2 minutes and centrifuged at 10000 r·min^-1^ for 10 minutes. Transfer the supernatant to a new EP tube. The juvenile hormone and molting hormone titers in each generation were monitored by Enzyme-Linked Immunosorbent Assay (ELISA) using insect juvenile hormone ELISA kit and insect molting hormone ELISA kit.

### Data statistics and analysis

The LC_50_ and LC_30_ values of imidacloprid and thiamethoxam were determined using the concentration–mortality regression line and a log-probit model of SPSS (version 23.0, SPSS Inc., Chicago, IL, USA). The activities of peroxidase, pyruvate kinase and trehalase of the 4^th^ instar nymphs and the expression levels of juvenile hormone and molting hormone of soybean aphids were analyzed by ANOVA combined with Duncan multiple range tests (DMRT), and least significant difference (LSD) method in each generation (F1-F5). For all treatments, *P* < 0.05 was considered significant. All figures were plotted using SigmaPlot 12.0.

## Results

### The toxicity of imidacloprid and thiamethoxam to 4^th^ instar nymphs of *A*. *glycines*

The toxicity of imidacloprid and thiamethoxam to 4^th^ instar nymphs was evaluated by concentration-mortality regression lines and a log-probit model (Table 1). The LC_50_ values of imidacloprid and thiamethoxam were estimated at 5.699 mg a.i./L and 10.548 mg a.i./L and the LC_30_ values were estimated at 3.451 mg a.i./L and 6.228 mg a.i./L, respectively.

**Table 1:** Toxicity of imidacloprid and thiamethoxam to the 4^th^ instar nymphs.

Insecticide	LC_50_ (mg a.i./L)	95% Confidence interval	LC_30_ (mg a.i./L)	95% Confidence interval	Slope ± SE^†^	χ^2^ (df)
Imidacloprid	5.699	4.458–7.296	3.451	2.479–4.415	2.408 ± 0.346	0.127 (5)
Thiamethoxam	10.548	8.176–13.602	6.228	4.374–8.046	2.292 ± 0.339	0.164 (5)

^†^SE = Standard error

### Effects of multi-generation imidacloprid and thiamethoxam stress on the activity of peroxidase of the 4^th^ instar nymphs in each generation (F1-F5)

**Fig 1:**
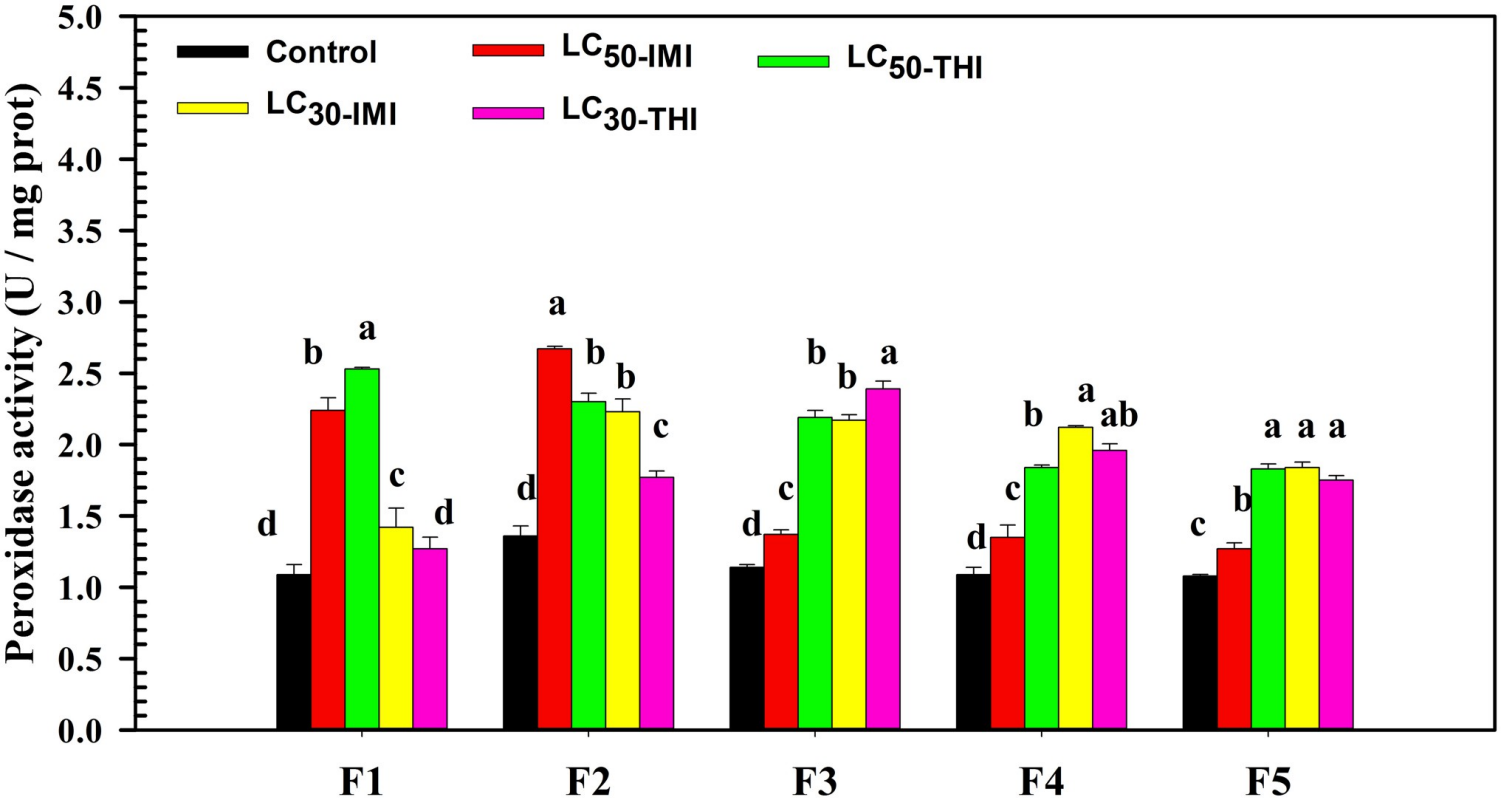
Changes in the peroxidase activity of soybean aphids in the same generation under imidacloprid and thiamethoxam stresses. Note: Different lowercase letters indicate that the peroxidase activity of soybean aphids in the same generation under imidacloprid and thiamethoxam stresses is significantly different (*P* < 0.05). F1, F2, F3, F4, F5 = The first, second, third, fourth, and fifth generation of the fourth instar nymphs, respectively. LC_50_-IMI represents the fourth instar nymphs treated with LC_50_ imidacloprid; LC_50_-THI represents the fourth instar nymphs treated with LC_50_ thiamethoxam; LC_30_-IMI represents the fourth instar nymphs treated with LC_30_ imidacloprid; LC_30_-THI represents the fourth instar nymphs treated with LC_30_ thiamethoxam.

Under continuous stress of imidacloprid and thiamethoxam, the activity of peroxidase in soybean aphids of F1-F5 generation increased first and then decreased (Fig 1). In F1 generation, LC_50_ thiamethoxam (F = 11.051, df = 2.01, *P* = 0.007) had the strongest stimulating effect on peroxidase activity and LC_30_ thiamethoxam (F = 3.846, df = 4, *P* = 0.233) had the weakest effect on peroxidase activity. Under the stress of LC_50_ thiamethoxam, the peroxidase activity in soybean aphids was the highest, which was 2.32 times that of the control. There was no significant difference in peroxidase activity between LC_30_ thiamethoxam (F = 3.846, df = 4, *P* = 0.233) treatment group and control group (*P >* 0.05). In F2 generation, LC_50_ imidacloprid (F = 6.724, df = 4, *P* = 0.000) stimulated peroxidase activity the most and LC_30_ thiamethoxam (F = 1.078, df = 4, *P* = 0.001) had the weakest effect on it. There was no significant difference between LC_50_ thiamethoxam (F = 0.180, df = 4, *P* = 0.000) and LC_30_ imidacloprid (F = 0.515, df = 4, *P* = 0.000) in stimulating peroxidase activity (*P >* 0.05). In F3 generation, LC_30_ thiamethoxam (F = 3.534, df = 4, *P* = 0.000) had the strongest activation effect on peroxidase activity. However, LC_50_ imidacloprid (F = 1.139, df = 4, *P* = 0.001) had the weakest effect on peroxidase activity. There was still no significant difference between LC_50_ thiamethoxam (F = 3.004, df = 4, *P* = 0.000) and LC_30_ imidacloprid (F = 2.306, df = 4, *P* = 0.000) in stimulating peroxidase activity (*P >* 0.05). In F4 generation, LC_30_ imidacloprid (F = 5.998, df = 4, *P* = 0.000) had the strongest activation effect on peroxidase activity. However, LC_50_ imidacloprid (F = 6.371, df = 4, *P* = 0.132) had the weakest effect on peroxidase activity. Different from F3 generation, the activation effect of LC_50_ thiamethoxam (F = 4.267, df = 4, *P* = 0.000) on enzyme activity was significantly weaker than that in LC_30_ imidacloprid treatment group (*P* < 0.05). In F5 generation, LC_50_ imidacloprid (F = 8.074, df = 2.280, *P* = 0.011) had the weakest activation effect on peroxidase activity. The activation effect of LC_50_ thiamethoxam (F = 10.127, df = 2.017, *P* = 0.016), LC_30_ imidacloprid (F = 3.532, df = 4, *P* = 0.000) and LC_30_ thiamethoxam (F = 1.447, df = 4, *P* = 0.000) on enzyme activity was significantly higher than that in LC_50_ imidacloprid treatment group (*P* < 0.05), but there was no significant difference among the three (*P >* 0.05).

### Effects of multigenerational imidacloprid and thiamethoxam stress on the activity of pyruvate kinase of the 4^th^ instar nymphs in each generation (F1-F5)

**Fig 2:**
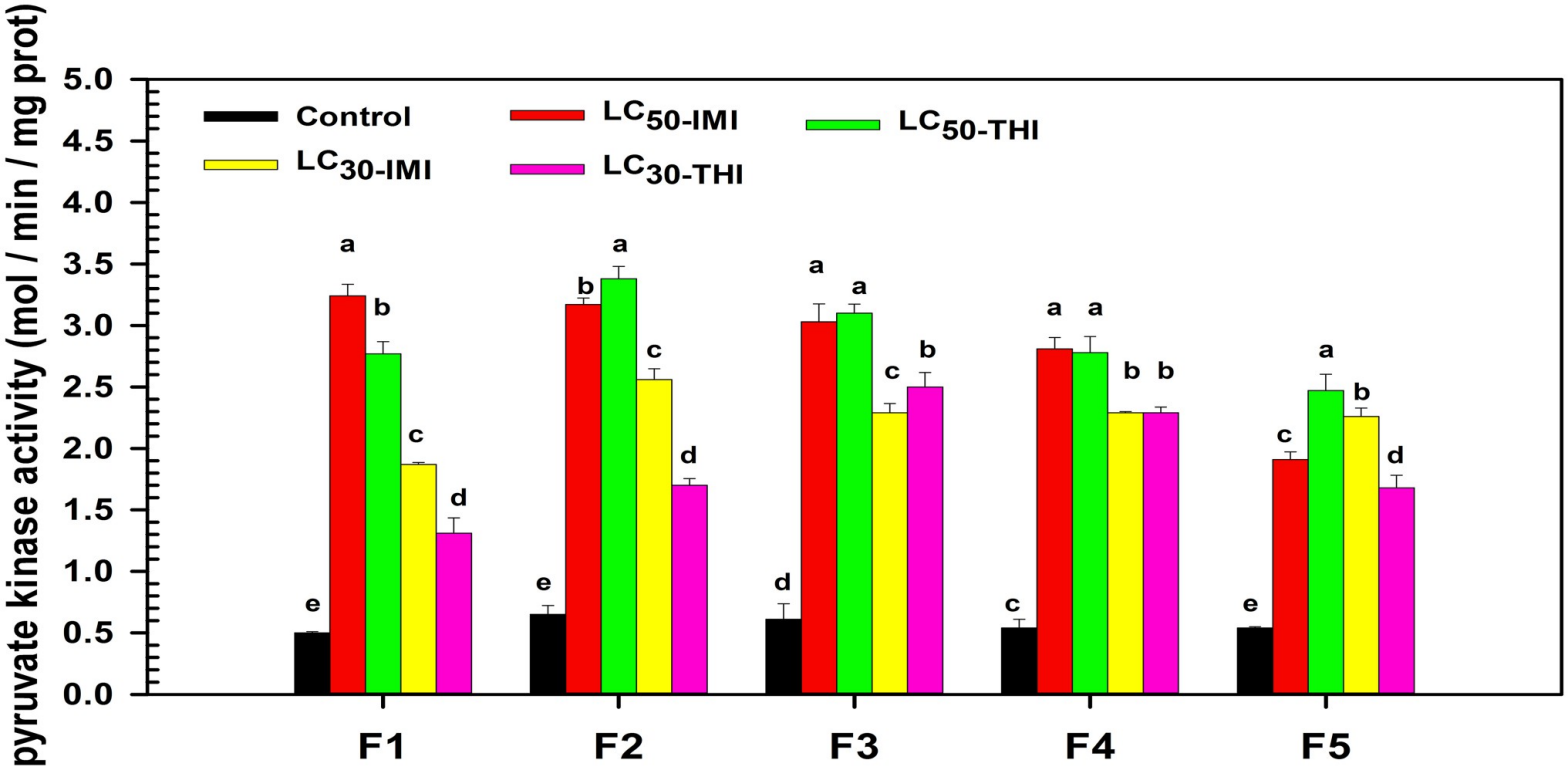
Changes of pyruvate kinase activity of soybean aphids in the same generation under imidacloprid and thiamethoxam stresses. Note: Different lowercase letters indicate that pyruvate kinase activity of soybean aphids in the same generation under imidacloprid and thiamethoxam stresses, respectively is significantly different (*P* < 0.05). F1, F2, F3, F4, F5 = The first, second, third, fourth, and fifth generation of the fourth instar nymphs, respectively. LC_50_-IMI represents the fourth instar nymphs treated with LC_50_ imidacloprid; LC_50_-THI represents the fourth instar nymphs treated with LC_50_ thiamethoxam; LC_30_-IMI represents the fourth instar nymphs treated with LC_30_ imidacloprid; LC_30_-THI represents the fourth instar nymphs treated with LC_30_ thiamethoxam.

The pyruvate kinase activity of the 4^th^ instar nymphs was significantly higher than that of the control group in each generation (F1-F5) under continuous stress of LC_50_ and LC_30_ of imidacloprid and thiamethoxam (*P* < 0.05) (Fig 2). In F1 generation, LC_50_ imidacloprid (F = 5.105, df = 4, *P* = 0.000) had the strongest stimulating effect on pyruvate kinase activity, while LC_30_ thiamethoxam (F = 8.746, df = 2.029, *P* = 0.008) had the weakest. The activation effect of LC_50_ thiamethoxam (F = 6.861, df = 4, *P* = 0.000) was significantly stronger than that in LC_30_ imidacloprid (F = 0.754, df = 4, *P* = 0.000) treatment group (*P* < 0.05). In F2 generation, LC_50_ thiamethoxam (F = 0.708, df = 4, *P* = 0.000) stimulated pyruvate kinase activity the most, which was different from F1 generation. LC_30_ thiamethoxam (F = 0.308, df = 4, *P* = 0.000) still had the weakest stimulating effect on enzyme activity, and that in soybean aphid was 1.7 mol/min/mg prot, which was 2.62 times that of the control. The activation effect of LC_50_ imidacloprid (F = 0.464, df = 4, *P* = 0.000) on enzyme activity was significantly stronger than LC_30_ imidacloprid (F = 0.083, df = 4, *P* = 0.000) treatment group (*P* < 0.05). In F3 generation, LC_50_ imidacloprid (F = 0.171, df = 4, *P* = 0.000) and thiamethoxam (F = 1.638, df = 4, *P* = 0.000) stimulated pyruvate kinase activity the most, and there was no significant difference between them (*P* > 0.05). The activation effect of LC_30_ imidacloprid (F = 1.507, df = 4, *P* = 0.000) on enzyme activity was weaker than that in LC_30_ thiamethoxam (F = 0.029, df = 4, *P* = 0.000) treatment group (*P* < 0.05). In F4 generation, LC_50_ imidacloprid (F = 0.643, df = 4, *P* = 0.000) and thiamethoxam (F = 2.460, df = 4, *P* = 0.000) still had the strongest stimulating effect on pyruvate kinase activity, and there was no significant difference between them (*P* > 0.05). This was consistent with the trend of the F3 generation. In addition, LC_30_ imidacloprid (F = 5.023, df = 4, *P* = 0.000) and thiamethoxam (F = 0.570, df = 4, *P* = 0.000) had the weakest stimulating effect on enzyme activity, and there was no significant difference between them (*P* > 0.05). In F5 generation, LC_50_ thiamethoxam (F = 13.449, df = 2.024, *P* = 0.001) had the strongest stimulating effect on pyruvate kinase activity, while LC_30_ thiamethoxam (F = 5.891, df = 4, *P* = 0.000) had the weakest stimulating effect. The activation effect of LC_30_ imidacloprid (F = 8.487, df = 2.090, *P* = 0.000) on enzyme activity was significantly stronger than LC_50_ imidacloprid (F = 2.289, df = 4, *P* = 0.000) treatment group (*P* < 0.05).

### Effects of multigenerational imidacloprid and thiamethoxam stress on the activity of trehalase of the 4^th^ instar nymphs in each generation (F1-F5)

**Fig 3:**
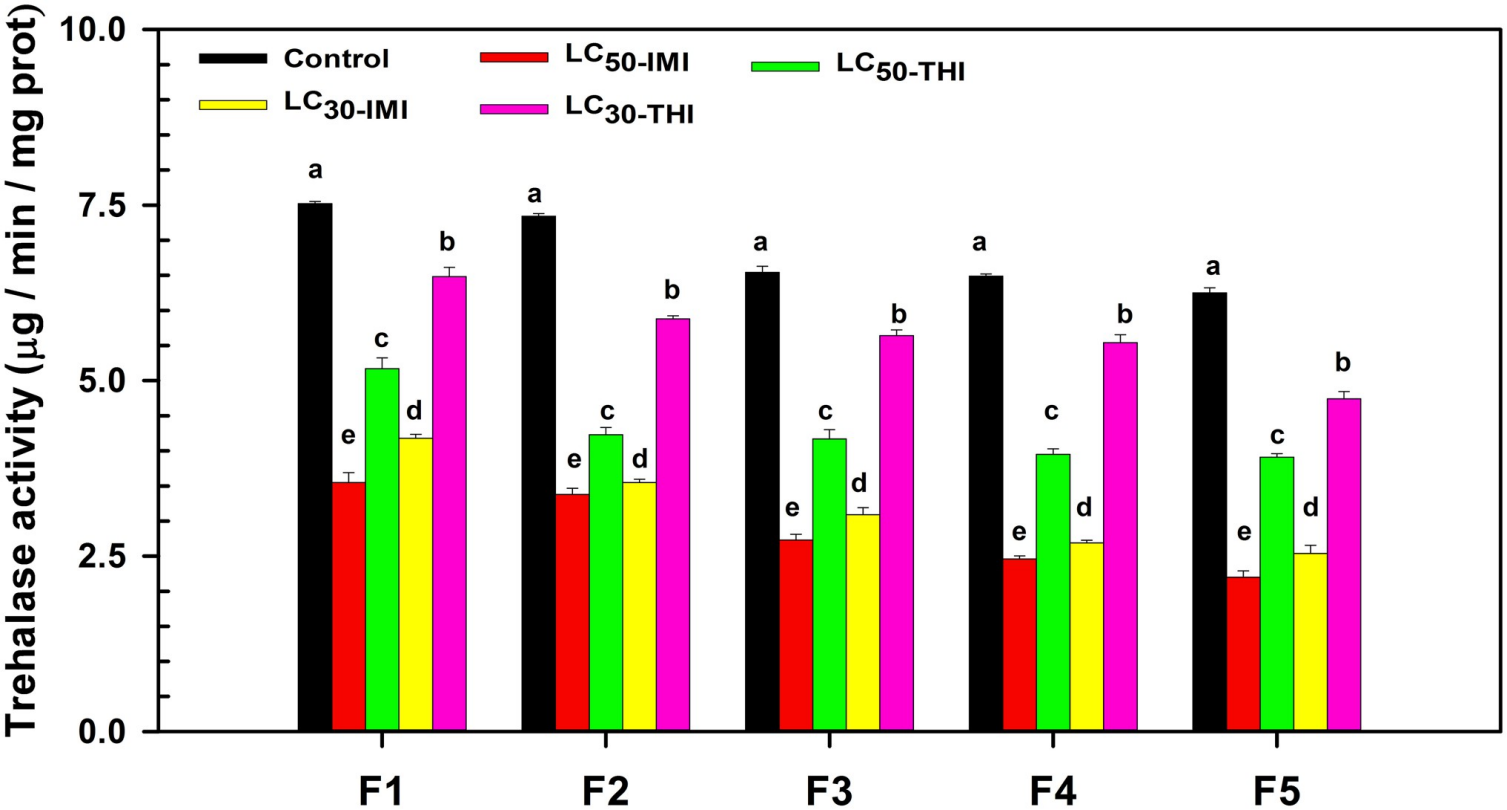
Changes of trehalase activity of soybean aphids in the same generation under imidacloprid and thiamethoxam stresses. Note: Different lowercase letters indicate that trehalase activity of soybean aphids in the same generation under imidacloprid and thiamethoxam stress is significantly different (*P* < 0.05). F1, F2, F3, F4, F5 = The first, second, third, fourth, and fifth generation of the fourth instar nymphs, respectively. LC_50_-IMI represents the fourth instar nymphs treated with LC_50_ imidacloprid; LC_50_-THI represents the fourth instar nymphs treated with LC_50_ thiamethoxam; LC_30_-IMI represents the fourth instar nymphs treated with LC_30_ imidacloprid; LC_30_-THI represents the fourth instar nymphs treated with LC_30_ thiamethoxam.

The trehalase activity of the 4^th^ instar nymphs under continuous exposure of LC_50_ and LC_30_ of imidacloprid and thiamethoxam was significantly lower than that of the control group in each generation (F1-F5) (Fig 3). In F1 generation, LC_30_ thiamethoxam (F = 7.823, df = 2.232, *P* = 0.004) had the weakest inhibitory effect on enzyme activity, while LC_50_ imidacloprid (F = 5.965, df = 4, *P* = 0.000) had the strongest inhibitory effect. In addition, the inhibitory effect of LC_50_ thiamethoxam (F = 3.399, df = 4, *P* = 0.000) on enzyme activity was significantly weaker than that in LC_30_ imidacloprid (F = 1.919, df = 4, *P* = 0.000) treatment group. In F1 generation, the inhibition degree of trehalase activity in each treatment group from strong to weak was LC_50_ imidacloprid, LC_30_ imidacloprid, LC_50_ thiamethoxam, LC_30_ thiamethoxam. In F2, F3, F4, and F5 generations, the effect of each treatment group on enzyme activity was consistent with the trend of F1 generation.

### Effects of multigenerational imidacloprid or thiamethoxam stress on the expression of juvenile hormone in soybean aphids of each generation

The expression of juvenile hormone in soybean aphids was significantly higher than the control group in each generation (F1-F5) following exposure to imidacloprid and thiamethoxam (*P* < 0.05) (Table 2). In F1 generation, the titer of juvenile hormone in the first (F = 0.714, df = 4, *P* = 0.000) and second (F = 0.104, df = 4, *P* = 0.000) instar nymphs was significantly higher than that in LC_50_ imidacloprid treatment groups under the stress of LC_30_ imidacloprid. This trend was also observed in the thiamethoxam treatment group. Under LC_30_ imidacloprid stress, the titer of juvenile hormone in the third (F = 0.196, df = 4, *P* = 0.000) and fourth (F = 3.100, df = 4, *P* = 0.000) instar nymphs and adults (F = 0.008, df = 4, *P* = 0.000) was significantly lower than that in LC_50_ imidacloprid treatment group. Under LC_30_ thiamethoxam stress, the juvenile hormone titer in the third (F = 1.459, df = 4, *P* = 0.000) instar nymphs and adults (F = 1.094, df = 4, *P* = 0.000) was significantly higher than that in LC_50_ thiamethoxam treatment group, while the change trend of hormone titer in the fourth (F = 3.119, df = 4, *P* = 0.000) instar nymphs was opposite to the former. In F2 generation, the change trend of juvenile hormone titer in the first and second instar nymphs of all treatment groups was the same as that in F1 generation. Under LC_30_ imidacloprid stress, the juvenile hormone titer in the third (F = 0.752, df = 4, *P* = 0.000) and fourth (F = 0.782, df = 4, *P* = 0.000) instar nymphs was significantly lower than that in the LC_50_ imidacloprid treatment group, while the hormone titer in the adults (F = 6.852, df = 4, *P* = 0.000) showed an opposite trend. Under LC_30_ thiamethoxam stress, the juvenile hormone titer in the third (F = 5.737, df = 4, *P* = 0.000) instar nymphs and adults (F = 1.000, df = 4, *P* = 0.000) was significantly higher than that in LC_50_ thiamethoxam treatment group, while the hormone titer in the fourth (F = 0.968, df = 4, *P* = 0.001) instar nymphs showed an opposite trend to the former. In F3 generation, the titer of juvenile hormone in the first (F = 0.315, df = 4, *P* = 0.000) instar nymphs treated with LC_30_ imidacloprid was significantly lower than that treated with LC_50_ imidacloprid. On the contrary, the hormone titer in the second (F = 1.559, df = 4, *P* = 0.000), third (F = 12.071, df = 2.020, *P* = 0.000) and fourth (F = 1.190, df = 4, *P* = 0.000) instar nymphs and adults (F = 0.869, df = 4, *P* = 0.000) was significantly higher than that in LC_50_ imidacloprid treatment group. The titer of juvenile hormone in the first (F = 5.451, df = 4, *P* = 0.000), second (F = 0.292, df = 4, *P* = 0.002) instar nymphs and adults (F = 0.107, df = 4, *P* = 0.000) in LC_30_ thiamethoxam treatment group was significantly lower than that in LC_50_ thiamethoxam treatment group. The titer of juvenile hormone in the third (F = 0.502, df = 4, *P* = 0.000) and fourth (F = 0.143, df = 4, *P* = 0.017) instar nymphs was significantly higher than that in LC_50_ thiamethoxam treatment group. In F4 generation, the juvenile hormone titer in the first (F = 1.312, df = 4, *P* = 0.000), second (F = 0.035, df = 4, *P* = 0.001) and third (F = 3.306, df = 4, *P* = 0.000) instar nymphs and adults (F = 0.164, df = 4, *P* = 0.000) in LC_30_ imidacloprid treatment group was significantly lower than that in LC_50_ imidacloprid treatment group. Only the hormone titer in the fourth (F = 0.005, df = 4, *P* = 0.000) instar nymphs was significantly higher than that in LC_50_ imidacloprid treatment group. The hormone titer in the first (F = 0.868, df = 4, *P* = 0.000), second (F = 2.459, df = 4, *P* = 0.018) and third (F = 0.865, df = 4, *P* = 0.000) instar nymphs of LC_30_ thiamethoxam treatment group was significantly higher than that in LC_50_ thiamethoxam treatment group, while the fourth (F = 0.148, df = 4, *P* = 0.000) instar nymphs and adults (F = 0.865, df = 4, *P* = 0.000) of LC_30_ thiamethoxam treatment group was significantly lower than that in LC_50_ thiamethoxam treatment group. In the F5 generation, the juvenile hormone titer of aphids treated with LC_30_ imidacloprid in the first (F = 0.510, df = 4, *P* = 0.001), second (F = 2.598, df = 4, *P* = 0.000) instar nymphs and adults (F = 0.188, df = 4, *P* = 0.000) was significantly lower than that treated with LC_50_ imidacloprid, while it in the third (F = 0.291, df = 4, *P* = 0.002) and fourth (F = 7.931, df = 2.228, *P* = 0.011) instar nymphs was significantly higher than that treated with LC_50_ imidacloprid. The juvenile hormone titer in the first (F = 0.037, df = 4, *P* = 0.026) and fourth (F = 0.525, df = 4, *P* = 0.000) instar nymphs of LC_30_ thiamethoxam treatment group was significantly lower than that in LC_50_ thiamethoxam treatment group, while the hormone titer in the second (F = 0.154, df = 4, *P* = 0.003) and third (F = 1.478, df = 4, *P* = 0.000) instar nymphs of LC_30_ thiamethoxam treatment group was significantly higher than that in LC_50_ thiamethoxam treatment group (Table 2).

**Table 2:** Effects of imidacloprid and thiamethoxam on the expression of juvenile hormone of soybean aphids in each generation.

Different generations	Different instars		LC_50_		LC_30_	
		Control	Imidacloprid	Thiamethoxam	Imidacloprid	Thiamethoxam
		Mean ± SE	Mean ± SE	Mean ± SE	Mean ± SE	Mean ± SE
		(ng / L)	(ng / L)	(ng / L)	(ng / L)	(ng / L)
F1	L1	61.29 ± 0.27 e	166.82 ± 0.29 c	112.17 ± 0.40 d	271.44 ± 0.48 b	341.88 ± 0.44 a
F1	L2	59.04 ± 0.13 e	124.52 ± 0.35 b	83.28 ± 0.22 d	137.72 ± 0.62 a	116.54 ± 0.17 c
F1	L3	52.30 ± 0.24 e	105.34 ± 0.15 b	81.68 ± 0.33 d	93.47 ± 0.36 c	150.05 ± 0.15 a
F1	L4	29.67 ±0.17 e	96.09 ± 0.08 a	80.30 ± 0.57 b	70.37 ± 0.37 c	69.26 ± 0.19 d
F1	Adult	18.12 ± 0.14 e	81.74 ± 0.34 b	69.07 ± 0.35 c	51.30 ± 0.39 d	98.29 ± 0.32 a
F2	L1	60.96 ± 0.06 e	165.80 ± 0.34 c	112.81 ± 0.60 d	277.21 ± 0.69 b	291.70 ± 0.27 a
F2	L2	52.28 ± 0.19 e	130.90 ± 0.38 b	92.63 ± 0.48 d	140.31 ± 0.53 a	117.95 ± 0.56 c
F2	L3	46.01 ± 0.12 e	114.33 ± 0.37 a	74.86 ± 0.21 d	100.10 ± 0.71 c	102.16 ± 0.62 b
F2	L4	17.71 ± 0.40 e	99.13 ± 0.43 a	69.67 ± 0.73 c	90.44 ± 0.60 b	65.21 ± 0.51 d
F2	Adult	16.45 ± 0.57 e	87.95 ± 0.76 c	63.70 ± 0.42 d	105.78 ± 0.22 a	94.55 ± 0.62 b
F3	L1	60.15 ± 0.46 e	190.01 ± 0.58 a	116.71 ± 0.36 c	142.49 ± 0.82 b	105.83 ± 0.99 d
F3	L2	48.25 ± 0.56 e	135.18 ± 0.62 b	94.10 ± 0.48 c	184.05 ± 0.26 a	90.98 ± 0.61 d
F3	L3	42.49 ± 0.63 e	114.41 ± 0.39 b	46.71 ± 0.46 d	178.05 ± 0.03 a	84.82 ± 0.36 c
F3	L4	16.95 ± 0.21 e	101.16 ± 0.62 b	42.59 ± 0.60 d	175.69 ± 0.32 a	44.38 ± 0.50 c
F3	Adult	15.52 ± 0.60 e	87.03 ± 0.51 b	53.82 ± 0.72 c	180.62 ± 0.73 a	43.25 ± 0.60 d
F4	L1	59.29 ± 0.37 e	187.82 ± 0.53 a	61.15 ± 0.53 d	139.16 ± 0.29 b	85.01 ± 0.33 c
F4	L2	45.71 ± 0.39 e	134.63 ± 0.56 a	76.16 ± 0.15 d	130.66 ± 0.55 b	77.86 ± 0.74 c
F4	L3	34.76 ± 0.96 e	114.38 ± 0.22 a	45.03 ± 0.74 d	97.17 ± 0.55 b	63.89 ± 0.41 c
F4	L4	16.58 ± 0.55 e	81.28 ± 0.43 b	42.55 ± 0.56 c	93.94 ± 0.40 a	31.34 ± 0.50 d
F4	Adult	14.11 ± 0.36 e	86.66 ± 0.40 a	50.17 ± 0.23 c	81.11 ± 0.52 b	31.16 ± 0.35 d
F5	L1	53.58 ± 0.85 e	141.16 ± 0.44 a	58.48 ± 0.52 c	136.93 ± 0.63 b	56.93 ± 0.58 d
F5	L2	45.36 ± 0.20 e	131.90 ± 0.11 a	68.07 ± 0.32 d	120.49 ± 0.20 b	71.92 ± 0.27 c
F5	L3	34.97 ± 0.39 e	89.06 ± 0.11 b	45.31 ± 0.23 d	91.22 ± 0.25 a	63.20 ± 0.27 c
F5	L4	15.98 ± 0.16 e	80.38 ± 1.13 b	39.14 ± 0.15 c	86.90 ± 0.13 a	28.81 ± 0.32 d
F5	Adult	14.17 ± 0.23 e	86.93 ± 0.21 a	44.82 ± 0.18 c	65.83 ± 0.21 b	30.56 ± 0.63 d

Different lowercase letters of the same row in the table indicate significant differences on juvenile hormone titer, and the significance level is 0.05. F1, F2, F3, F4, F5 = The first, second, third, fourth, and fifth generation. L1, L2, L3, L4, L5 = The first, second, third, fourth, and fifth instar nymphs. The titer unit of juvenile hormone is “ng / L”.

### Effects of multigenerational imidacloprid or thiamethoxam stress on the expression of molting hormone in soybean aphids of each generation (F1-F5)

LC_50_ imidacloprid and LC_50_ thiamethoxam inhibited the expression of molting hormone in soybean aphids of each generation. LC_30_ imidacloprid or LC_30_ thiamethoxam significantly stimulated the expression of molting hormone in the 1^st^ and 2^nd^ instar nymphs in each generation (*P* < 0.05) (Table 3). In F1 generation, there was no significant difference between LC_50_ imidacloprid and LC_50_ thiamethoxam treatment on inhibiting the expression of molting hormone in the first (F = 0.005, df = 4, *P* = 0.008) instar nymphs. The inhibitory effect of LC_50_ imidacloprid on hormone expression in the second (F = 6.064, df = 4, *P* = 0.000), third (F = 1.139, df = 4, *P* = 0.000) and fourth (F = 3.426, df = 4, *P* = 0.000) instar nymphs and adults (F = 1.233, df = 4, *P* = 0.000) was significantly weaker than that in LC_50_ thiamethoxam treatment group. The promotion effect of LC_30_ imidacloprid stress on hormone expression was significantly stronger than that in LC_30_ thiamethoxam treatment group. In F2 generation, the stress of LC_50_ thiamethoxam significantly inhibited the hormone expression of soybean aphid at all instars than that in LC_50_ imidacloprid treatment group. The promotion effect of LC_30_ imidacloprid stress on hormone expression in the first (F = 0.032, df = 4, *P* = 0.000), second (F = 0.009, df = 4, *P* = 0.000), third (F = 13.495, df = 2.014, *P* = 0.045) instar nymphs and adults (F = 10.891, df = 2.105, *P* = 0.029) was significantly stronger than that in LC_30_ thiamethoxam treatment group. On the contrary, the hormone expression in the fourth (F = 4.401, df = 4, *P* = 0.006) instar nymphs was significantly weaker than that in LC_30_ thiamethoxam treatment group. In F3 generation, the inhibition degree of LC_50_ thiamethoxam stress on hormone expression of all instars soybean aphid was still significantly stronger than that in LC_50_ imidacloprid treatment group. The promotion effect of LC_30_ imidacloprid stress on hormone expression in aphids from the first to the fourth instars was significantly stronger than that in LC_30_ thiamethoxam treatment group. And the hormone expression in adults (F = 0.177, df = 4, *P* = 0.000) was significantly weaker than that in LC_30_ thiamethoxam treatment group. In F4 generations, the inhibition of LC_50_ imidacloprid on hormone expression in the first (F = 0.119, df = 4, *P* = 0.001) instar nymphs and adults (F = 9.483, df = 2.138, *P* = 0.006) was significantly stronger than that in the LC_50_ thiamethoxam treatment group, while the inhibition of hormone titer in the second (F = 0.009, df = 4, *P* = 0.003), third (F = 0.566, df = 4, *P* = 0.004) and fourth (F = 11.035, df = 2.043, *P* = 0.001) instar nymphs was significantly weaker than that in the LC_50_ thiamethoxam treatment group. The promotion effect of LC_30_ imidacloprid stress on the hormone expression in the first (F = 2.464, df = 4, *P* = 0.026), third (F = 0.542, df = 4, *P* = 0.025) and fourth (F = 4.071, df = 4, *P* = 0.121) instar nymphs was significantly stronger than that in LC_30_ thiamethoxam treatment group, while it in the second (F = 0.222, df = 4, *P* = 0.006) instar nymphs and adults (F = 0.371, df = 4, *P* = 0.002) was significantly weaker than that in LC_30_ thiamethoxam treatment group. In the F5 generation, LC_50_ imidacloprid significantly inhibited the expression of molting hormone in the first (F = 3.384, df = 4, *P* = 0.000), second (F = 5.830, df = 4, *P* = 0.000), third (F = 11.032, df = 2.101, *P* = 0.009) instar nymphs and adults (F = 1.412, df = 4, *P* = 0.000), which compared with LC_50_ thiamethoxam treatment group. The inhibition of the hormone expression in the fourth (F = 8.806, df = 2.175, *P* = 0.001) instar nymphs was significantly weaker than that in LC_50_ thiamethoxam treatment group. The promotion effect of LC_30_ imidacloprid stress on the hormone expression in the first (F = 3.512, df = 4, *P* = 0.109) instar nymphs was significantly weaker than that in LC_30_ thiamethoxam treatment group, while it in the second (F = 2.610, df = 4, *P* = 0.004), third (F = 5.681, df = 4, *P* = 0.037) and fourth (F = 2.750, df = 4, *P* = 0.018) instar nymphs was significantly stronger than that in LC_30_ thiamethoxam treatment group (Table 3).

**Table 3:** Effects of imidacloprid and thiamethoxam on the expression of molting hormone of soybean aphids in each generation.

Different generations	Different instars		LC_50_		LC_30_	
		Control	Imidacloprid	Thiamethoxam	Imidacloprid	Thiamethoxam
		Mean ± SE	Mean ± SE	Mean ± SE	Mean ± SE	Mean ± SE
		(ng / L)	(ng / L)	(ng / L)	(ng / L)	(ng / L)
F1	L1	25.62 ± 0.51 c	22.14 ± 0.09 d	21.78 ± 0.09 d	75.01 ± 0.29 a	26.20 ± 0.17 b
F1	L2	24.59 ± 0.18 c	21.17 ± 0.05 d	15.66 ± 0.20 e	95.28 ± 0.26 a	26.28 ± 0.03 b
F1	L3	21.73 ± 0.09 c	21.30 ± 0.40 c	18.19 ± 0.23 d	30.95 ± 0.32 a	23.69 ± 0.52 b
F1	L4	19.46 ± 0.52 c	18.25 ± 0.49 d	7.64 ± 0.07 e	24.66 ± 0.35 a	21.73 ± 0.39 b
F1	Adult	16.99 ± 0.19 c	16.03 ± 0.06 d	15.34 ± 0.02 e	21.79 ± 0.14 a	20.36 ± 0.04 b
F2	L1	23.80 ± 0.24 c	22.42 ± 0.31 d	16.74 ± 0.43 e	30.68 ± 0.37 a	25.23 ± 0.42 b
F2	L2	24.20 ± 0.42 c	21.41 ± 0.30 d	12.65 ± 0.47 e	45.81 ± 0.20 a	26.01 ± 0.21 b
F2	L3	22.65 ± 0.14 c	19.67 ± 0.24 d	8.89 ± 0.40 e	25.61 ± 0.77 a	23.61 ± 0.05 b
F2	L4	18.53 ± 0.52 c	16.61 ± 0.03 d	7.51 ± 0.18 e	21.15 ± 0.19 b	21.77 ± 0.08 a
F2	Adult	17.75 ± 0.36 c	16.71 ± 0.45 d	10.15 ± 0.37 e	21.12 ± 0.61 a	19.20 ± 0.10 b
F3	L1	23.91 ± 0.61 c	20.55 ± 0.17 d	15.75 ± 0.39 e	26.82 ± 0.40 a	25.51 ± 0.62 b
F3	L2	22.94 ± 0.63 b	19.54 ± 0.57 c	11.37 ± 0.20 d	25.17 ± 0.15 a	25.86 ± 0.19 a
F3	L3	21.99 ± 0.17 b	19.63 ± 0.47 c	8.99 ± 0.58 d	23.34 ± 0.04 a	22.10 ± 0.13 b
F3	L4	18.45 ± 0.55 c	16.82 ± 0.33 d	7.06 ± 0.02 e	21.00 ± 0.61 a	19.68 ± 0.05 b
F3	Adult	16.59 ± 0.34 c	12.03 ± 0.31 d	11.33 ± 0.21 e	17.95 ± 0.11 b	19.13 ± 0.10 a
F4	L1	23.94 ± 0.15 b	10.28 ± 0.77 d	14.73 ± 0.61 c	27.04 ± 0.10 a	26.26 ± 0.38 a
F4	L2	23.30 ± 0.34 c	13.33 ± 0.37 d	11.30 ± 0.39 e	24.63 ± 0.27 b	25.74 ± 0.24 a
F4	L3	21.22 ± 0.47 b	12.35 ± 0.76 c	9.19 ± 0.54 d	23.42 ± 0.54 a	22.04 ± 0.42 b
F4	L4	18.98 ± 0.70 b	14.00 ± 0.04 c	6.04 ± 0.38 d	20.37 ± 0.34 a	19.96 ± 0.13 a
F4	Adult	16.99 ± 0.18 b	6.37 ± 0.64 d	10.77 ± 0.12 c	16.46 ± 0.51 b	19.02 ± 0.39 a
F5	L1	23.49 ± 0.04 c	8.45 ± 0.31 e	14.77 ± 0.13 d	25.19 ± 0.15 b	25.65 ± 0.36 a
F5	L2	24.87 ± 0.12 c	9.44 ± 0.05 e	11.15 ± 0.14 d	25.79 ± 0.08 a	25.15 ± 0.16 b
F5	L3	22.48 ± 0.60 ab	5.05 ± 0.12 d	9.28 ± 0.73 c	23.22 ± 0.15 a	22.25 ± 0.52 b
F5	L4	18.57 ± 0.77 b	9.27 ± 0.07 c	3.52 ± 0.34 d	20.74 ± 0.30 a	19.27 ± 0.59 b
F5	Adult	17.27 ± 0.43 b	4.27 ± 0.23 e	11.36 ± 0.35 d	16.21 ± 0.27 c	18.46 ± 0.24 a

Different lowercase letters of the same row in the table indicate significant differences on molting hormone titer, and the significance level is 0.05. F1, F2, F3, F4, F5 = The first, second, third, fourth, and fifth generation. L1, L2, L3, L4, L5 = The first, second, third, fourth, and fifth instar nymphs. The titer unit of molting hormone is “ng / L”.

## Discussion

We found that continuous exposure to imidacloprid and thiamethoxam stimulated the activities of peroxidase and pyruvate kinase in the 4^th^ instar nymphs in each generation (F1-F5). Changes in enzyme activity might be linked to the regulation of insect metabolism against pesticides. Similarly, Sheng et al. found that increased resistance of wasps to phoxim and cypermethrin was accompanied by increased peroxidase activity [18]. Our study also found that the continuous exposure of LC_50_ thiamethoxam had a more substantial stimulating impact on the peroxidase activity of the 4^th^ instar nymphs than LC_50_ imidacloprid in F1, F3, F4, and F5 generations. This might be due to thiamethoxam decomposition into clothianidin after field application. Clothianidin did not decompose easily underground, and its duration was longer than thiamethoxam and imidacloprid, which further prolonged the stress time to soybean aphids. Long-term clothianidin stress might more strongly stimulate the antioxidant regulatory response in soybean aphids [13, 27]. Another factor explaining these findings is that imidacloprid and thiamethoxam have distinct water solubility and degradation rates. These characteristics could also cause a difference in their control ability and impact soybean aphids’ antagonism strategy to insecticides [28, 29]. At the same time, we found that peroxidase and pyruvate kinase activities in soybean aphids increased rapidly, first to peak and then decreased slowly generation by generation under imidacloprid and thiamethoxam stress. Continuous stress of imidacloprid or thiamethoxam might induce the rapid activation of antioxidant and anti-stress regulation of soybean aphids. With the enhancement of resistance regulation of soybean aphids, they gradually adapted to low-level stress. The body no longer needs to synthesize a large amount of antioxidant enzymes and consume a large amount of energy substances to resist the damage of insecticides. Hence, the enzyme activity gradually decreased generation by generation [14, 16, 30, 31]. Furthermore, we found that changes in peroxidase or pyruvate kinase did not accompany changes in trehalase activity. Both imidacloprid and thiamethoxam inhibited the activity of trehalase. The inhibition effect of imidacloprid on trehalase activity was stronger than thiamethoxam at the same concentration. The inhibition effects were sustained generation after generation. Inhibition of trehalase activity could directly affect blood glucose balance and carbohydrate metabolism in insects [32]. This might be affected the development of the insects’ ovaries and the maturation of their eggs and even further influence the development, molting, and other physiological processes [19, 20]. We hypothesized that pyruvate kinase and trehalase activities might be closely related to the dynamic regulation of juvenile and molting hormones.

Our results showed that inhibition of trehalase activity was accompanied by a low titer level of molting hormone under continuous exposure to LC_50_ imidacloprid. The phenomenon of mutual regulation between trehalase activity and molting hormone expression level was found to similar conclusions in the experiment of Tatun et al. injecting 20-hydroxyecdysone into *Omphisa fuscidentalis* Hampson (Lepidoptera: Pyralidae) [33]. In our study, LC_50_ imidacloprid and Thiamethoxam multigenerational stress-induced increased juvenile hormone titer and decreased molting hormone titer in soybean aphids. This was consistent with the results in the experiments of Yu et al. [34] using imidacloprid to stress *Chilo suppressalis* Walker (Lepidoptera: Pyralidae) and Ge et al. using deltamethrin and triazophos to stress the brown planthopper, *Nilaparvata lugens* Stl (Hemiptera: Delphacidae) [35]. Our research also found that different from the effects of LC_50_ imidacloprid and thiamethoxam, LC_30_ could affect the biosynthesis of molting hormone, leading to the disorder of hormone homeostasis, promoting molting, and stimulating the development of the 1^st^ and 2^nd^ instars nymphs. This phenomenon of stimulating growth with the low concentration of insecticides puts great pressure on the control of soybean aphids in the field [36].

Similarly, chronic multigenerational exposure to imidacloprid or thiamethoxam might diminish their susceptibility to soybean aphids. Long-term exposure to pesticides with a hormesis impact on pests may enhance pest resistance, which has significant implications for pest management. This phenomenon was observed only when the peach aphids *Myzus persicae* Sulzer.

(Hemiptera: Aphididae) were exposed to insecticides for multiple generations [37]. This finding supported the possibility that the adaptation of soybean aphids might be an intergenerational phenomenon under continuous insecticides stress. The characteristics of rapid growth, rapid reproduction, and frequent generation of soybean aphids could be combined to carry out multi-generation research when studying the effects of insecticides on these insects. These findings may be helpful in predicting multigenerational soybean aphid adaptation to imidacloprid and thiamethoxam and understanding the differences between the effects of these two insecticides. Moreover, this research was crucial in delaying the resistance development in soybean aphids, extending the insecticide’s service life, and efficiently controlling soybean aphids using imidacloprid and thiamethoxam. In future, we will continue to focus on the impacts of neonicotinoids on the transcriptome and associated resistance genes of soybean aphids and will provide critical information for their effective management.

## Conclusion

Our data demonstrated that peroxidase and pyruvate kinase activities could be stimulated for multiple generations under the stress of LC_30_ and LC_50_ imidacloprid or thiamethoxam. In contrast, the activities of trehalase were continuously inhabited. LC_30_ imidacloprid and thiamethoxam could affect molting hormone biosynthesis and stimulate the molting of 1^st^ and 2^nd^ instars nymphs. In addition, this study found that the adaptation of the soybean aphids to imidacloprid or thiamethoxam stress might be a multigenerational phenomenon. These results provided important data for effective control of the soybean aphids using imidacloprid and thiamethoxam.

## Supporting information

S1 Fig Changes in the activity of peroxidase of soybean aphids in the same generation under imidacloprid and thiamethoxam stresses. Note: Different lowercase letters indicate that the peroxidase activity of soybean aphids in the same generation under imidacloprid and thiamethoxam stresses is significantly different (*P* < 0.05). F1, F2, F3, F4, F5 = The first, second, third, fourth, and fifth generation of the fourth instar nymphs. LC_50_-IMI represents the fourth instar nymphs treated with LC_50_ imidacloprid; LC_50_-THI represents the fourth instar nymphs treated with LC_50_ thiamethoxam; LC_30_-IMI represents the fourth instar nymphs treated with LC_30_ imidacloprid; LC_30_-THI represents the fourth instar nymphs treated with LC_30_ thiamethoxam.
(XLS)

S2 Fig Changes of the activity of pyruvate kinase of soybean aphids in the same generation under imidacloprid and thiamethoxam stresses. Note: Different lowercase letters indicate that pyruvate kinase activity of soybean aphids in the same generation under imidacloprid and thiamethoxam stresses, respectively is significantly different (*P* < 0.05). F1, F2, F3, F4, F5 = The first, second, third, fourth, and fifth generation of the fourth instar nymphs. LC_50_-IMI represents the fourth instar nymphs treated with LC_50_ imidacloprid; LC_50_-THI represents the fourth instar nymphs treated with LC_50_ thiamethoxam; LC_30_-IMI represents the fourth instar nymphs treated with LC_30_ imidacloprid; LC_30_-THI represents the fourth instar nymphs treated with LC_30_ thiamethoxam.
(XLS)

S3 Fig Changes of trehalase activity of soybean aphids in the same generation under imidacloprid and thiamethoxam stresses. Note: Different lowercase letters indicate that trehalase activity of soybean aphids in the same generation under imidacloprid and thiamethoxam stress is significantly different (*P* < 0.05). F1, F2, F3, F4, F5 = The first, second, third, fourth, and fifth generation of the fourth instar nymphs. LC_50_-IMI represents the fourth instar nymphs treated with LC_50_ imidacloprid; LC_50_-THI represents the fourth instar nymphs treated with LC_50_ thiamethoxam; LC_30_-IMI represents the fourth instar nymphs treated with LC_30_ imidacloprid; LC_30_-THI represents the fourth instar nymphs treated with LC_30_ thiamethoxam.
(XLS)

S1 Table Toxicity of imidacloprid or thiamethoxam to the 4^th^ instar nymphs. SE = Standard error.
(XLS)

S2 Table Effects of imidacloprid and thiamethoxam on the expression of juvenile hormone of soybean aphids in each generation. Different lowercase letters of the same row in the table indicate significant differences on juvenile hormone titer, and the significance level is 0.05. F1, F2, F3, F4, F5 = The first, second, third, fourth, and fifth generation. L1, L2, L3, L4, L5 = The first, second, third, fourth, and fifth instar nymphs. The titer unit of juvenile hormone is “ng / L”.
(XLS)

S3 Table Effects of imidacloprid and thiamethoxam on the expression of molting hormone of soybean aphids in each generation. Different lowercase letters of the same row in the table indicate significant differences on molting hormone titer, and the significance level is 0.05. F1, F2, F3, F4, F5 = The first, second, third, fourth, and fifth generation. L1, L2, L3, L4, L5 = The first, second, third, fourth, and fifth instar nymphs. The titer unit of molting hormone is “ng / L”.
(XLS)
